# Atomic Layer Deposition of Pt Nanoparticles for Microengine with Promoted Catalytic Motion

**DOI:** 10.1186/s11671-016-1515-5

**Published:** 2016-06-13

**Authors:** Chi Jiang, Gaoshan Huang, Shi-Jin Ding, Hongliang Dong, Chuanling Men, Yongfeng Mei

**Affiliations:** School of Energy and Power Engineering, University of Shanghai for Science and Technology, Shanghai, 200093 China; Department of Materials Science, Fudan University, Shanghai, 200433 China; School of Microelectronics, Fudan University, Shanghai, 200433 China

**Keywords:** Microengine, Atomic layer deposition, Pt nanoparticles, Surface area

## Abstract

Nanoparticle-decorated tubular microengines were synthesized by a combination of rolled-up nanotechnology and atomic layer deposition. The presence of Pt nanoparticles with different sizes and distributions on the walls of microengines fabricated from bilayer nanomembranes with different materials results in promoted catalytic reaction efficiency, which leads to an ultrafast speed (the highest speed 3200 μm/s). The motion speed of the decorated microengines fits the theoretical model very well, suggesting that the larger surface area is mainly responsible for the acceleration of the motion speed. The high-speed nanoparticle-decorated microengines hold considerable promise for a variety of applications.

**Electronic supplementary material:**

The online version of this article (doi:10.1186/s11671-016-1515-5) contains supplementary material, which is available to authorized users.

## Background

The syntheses of micro-/nano-engines that are able to perform various tasks have attracted great attention with the development of nanotechnology. Among these artificial engines, catalytic micro-/nano-engines with different shapes of rod [1], sphere [2], helical [3], and tubes [4], mimicking their counterparts in the nature [5], are capable of moving autonomously in the presence of corresponding fuels or powered by various external stimuli such as light [6], magnetic [7], or ultrasound fields [8]. Particularly, bubble-propelled tubular microengines have become highly attractive due to their impressive features including high-power output, ultrafast movement speed, and independence of motion on ionic strength in liquid media [9]. In order to fabricate microtubular structures with catalytic inner surfaces, different methods have been employed, including template electrodeposition methods using porous membranes [10, 11] and roll-up technology [12]. Rolled-up technology have a few advantages like wide range of materials engaged and easy tuning of length and diameter [12], and the fabricated microengines have been applied to cargo-towing [13], tissue-drilling [14], dynamic assembly [15], and so on. With further development of micro-/nano-electromechanical system, powerful micro-/nano-engines with high speed and large driving force are demanded to accomplish complex tasks by overcoming the viscous force at low Reynolds number [16], and various measures have been applied to improve the performance of the catalytic microengines. For instance, graphene [17], carbon nanotube [18], and nanoparticles [19] have been used to promote catalytic reactions, and the hierarchical nanoporous microtubular engines [20] have been reported to improve fuel refilling. Although these methods can improve the performance of microengines and the motion speeds to some extent, the preparation process is relatively complicated and the poor utilization of the expensive Pt material is also an obvious drawback. There exists a need for scalable synthetic methods to coat the surface of the microengines with precise control of the catalyst distribution. Most importantly, the size distribution of nanoparticle and efficient loading of the noble-metal catalyst should be of great importance to improve the performance of microengines.

We consider that a convenient method to commendably satisfy the requirements may be the combination of rolled-up nanotechnology and atomic layer deposition (ALD). ALD has emerged as an important technique of depositing thin films for a variety of applications [21]. Sequential self-limiting surface reaction steps enable excellent thickness control, conformal coating on highly complex nanostructures, and good uniformity over a large area [22]. The ALD of noble metals such as Pt has been shown to generate well-dispersed nanoparticles during the initial stages of growth [23–26]. This feature could be meaningful for catalytic engines since the nanoparticle array with large surface area and high surface-area-to-volume ratio can effectively improve the utilization efficiency of catalyst [27].

Here, we demonstrate a simplified approach using ALD of fabricating Pt nanoparticles for the mass production of highly efficient microtubular engines. The presence of Pt nanoparticles with different sizes and distributions on the walls of microengines results in promoted catalytic reaction efficiency. Correspondingly, the Pt nanoparticle-decorated microengines exhibit significant speed acceleration compare to the theoretical speed of smooth microengines with the same diameter and length. The high performance of current Pt nanoparticle-decorated microengines offers a great opportunity for designing and producing ultrapowerful micro-/nanomachines for practical applications like cargo and drug delivery.

## Results and Discussion

### Fabrication of Pt Nanoparticle-Decorated Tubular Microengine

**Fig. 1:**
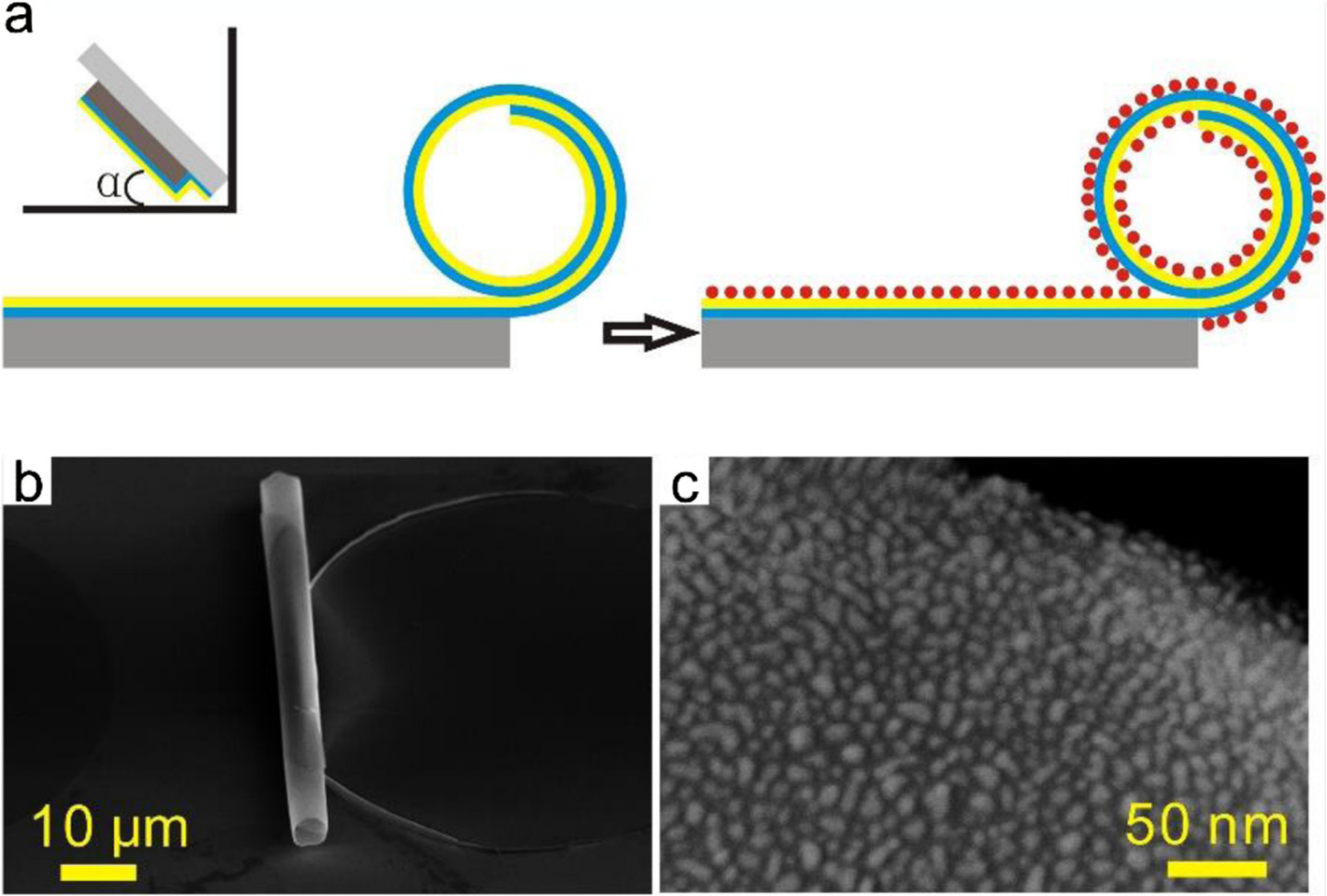
Fabrication of Pt nanoparticle-decorated tubular microengine. **a** Diagram of the fabrication procedure. **b** SEM image of a SiO/SiO_2_/Pt nanoparticle microtube. **c** An enlarged image of the Pt nanoparticles on the inner wall of the microtube

Figure 1a illustrates the experimental procedure for the fabrication of Pt nanoparticle-decorated microengine. The fabrication strategy was based on rolled-up technology using photoresist as a sacrificial layer (see the “Experimental Section” section for details) [12]. Briefly, bilayer nanomembranes with different thicknesses and thickness ratios (e.g., SiO/SiO_2_ 5/20 nm, Ti/SiO_2_ 20/10 nm, Ti/Co 10/10 nm, SiO_2_/Ti 10/20 nm) were deposited on photoresist patterns via electron beam evaporation. After selective etching of the sacrificial layer, the bilayer was set free and the strain gradient causes rolling of the bilayer nanomembrane into microtube [12]. Geometrical parameters such as the length, diameter, and shape of the microtubes can be tuned on one hand by changing the dimensions of the photoresist patterns and on the other hand by controlling the angles, rates, and thicknesses during the depositions of the nanomembranes [28]. After formation of microtubes, Pt nanoparticles were coated on the tube wall by ALD, where two self-limiting and complementary reactions are used in an alternating sequence [29]. On the first circle, the PtO_x_ was produced during piping in a pulse of O_2_. Then, a pulse of methylcyclopentadienyl-(trimethyl) platinum(IV) ((MeCp)Pt(Me)_3_) is forced into the generator’s chamber, which reacted with the PtO_x_ layer and O atoms are removed, leaving only Pt. On the next cycle, the unreacted precursor was removed and Pt surface was oxidized during the pulse of O_2_, preparing it for the next cycle [30, 31]. Due to its high surface energy, Pt deposition on supports proceeds via an island growth mechanism (Volmer–Weber mechanism) during the initial stages of ALD processes [30, 31]. Ultimately, after a sufficient number of exposure cycles, the islands will merge to form a film. However, for applications in catalysis, it is typically undesirable to obtain a continuous film: the island structure should be maintained because the islands/nanoparticles with a high surface-area-to-volume ratio should have better catalytic activity compared with flat layer [32, 33]. In current work, Pt nanoparticles were uniformly coated on the surface of the tube walls by precisely controlling the number of cycles adopted.

Figure 1b displays bird-view scanning electron microscopy (SEM) image of a typical 50-μm-long Pt nanoparticle-decorated SiO/SiO_2_ microtube under low magnification. A close examination of such tubular structure (Fig. 1c) reveals that, unlike common rolled-up microtube with a Pt smooth surface [34], the current microtube is covered by nanoparticles with average diameters of ~10 nm. As will be illustrated below, such Pt nanostructure leads to a dramatically increased catalytic surface area [35] and corresponding improved propulsion efficiency [36].

**Fig. 2:**
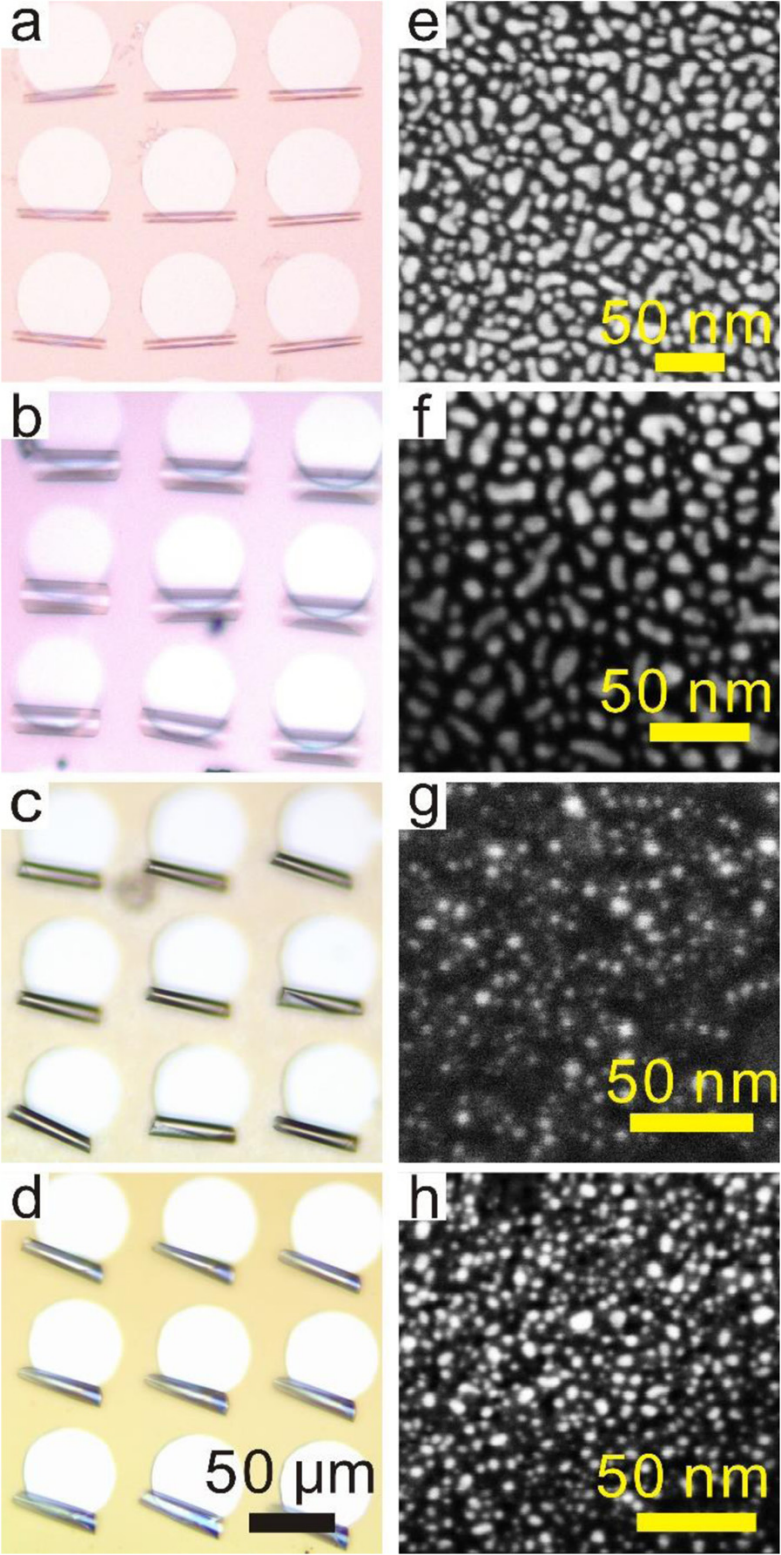
Optical images of microtube arrays made from different bilayers: **a** SiO/SiO_2_ (5/20 nm), **b** Ti/SiO_2_ (20/10 nm), **c** Ti/Co (10/10 nm), and **d** SiO_2_/Ti (10/20 nm). **e**–**h** Corresponding SEM images demonstrate the distribution of Pt nanoparticles on the inner tube walls

We further investigated formation of Pt nanoparticles on different microtubular structures. Figure 2a–d shows that the microtubes of well-defined lengths and geometries by rolling different nanomembranes can be arranged into ordered arrays. Such arrays can be mass-produced by normal photolithography and this makes it easier to prepare a large number of microengines simultaneously [37]. As demonstrated in our previous work [12], the diameter can be tuned by changing the layer thicknesses, the thickness ratios, and the built-in strain in the nanomembrane. In present case, the SiO/SiO_2_ microtubes have diameter of 5 μm and Ti/SiO_2_ microtubes have lager diameter of 12 μm due to different nanomembrane thicknesses and stress gradient therein. In order to illustrate the Pt nanoparticles on the inner tube wall for details, Fig. 2e–h shows the corresponding SEM images. It is found that nanometer-scale islands nucleate on the wall of microtubes after ALD cycles. The energy dispersive X-ray spectra of the samples (not shown) clearly prove the presence of Pt on the tube walls. However, the nanoparticles on different top layer of nanomembranes (inner tube wall) show different sizes and morphologies after the same ALD process. The nanomembranes with oxide top layers (i.e., SiO/SiO_2_ and Ti/SiO_2_ bilayers) exhibit very flat and smooth surface and the Pt nanoparticles on them appear in the form of irregular shapes like ellipses and bars (Fig. 2e, f). On the other hand, the nanomembrane containing metallic layers (Ti/Co and SiO_2_/Ti in present case) is relatively rough and uneven, and Pt nanoparticles in the form of small semi-spheres on the surface can be observed (Fig. 2g, h). We believe that the morphological difference in the bilayer nanomembranes is mainly due to different growth models and surface energies between oxide and metals during electron beam evaporation [38, 39]. In such incoherent growth condition, the growth of large particles/islands as a result of dissolution of small particles/islands can be explained by Ostwald ripening mechanism [38]. These factors also cause the change of the shapes of Pt nanoparticles when they are deposited on nanomembranes with oxide and metal top layer. However, it should be mentioned that, for the sake of simplicity, we suppose the nanoparticles are all in the shape of semi-spheres in the following model. This certainly introduces deviation in the model, but as we will discuss later, the experimental results can fit theoretical prediction well, suggesting that this simplicity is acceptable. Based on Fig. 2e–h, we have calculated the mean sizes of Pt nanoparticle on the inner wall (top layer). The results are 11, 10, 5, and 6 nm for nanoparticles on the surfaces of SiO/SiO_2_, Ti/SiO_2_, Ti/Co, and SiO_2_/Ti nanomembranes, respectively (Additional file 1: Figure S1). And the densities of nanoparticles are as high as 3.07 × 10^15^, 4.62 × 10^15^, and 1.85 × 10^16^, and 3.18 × 10^16^ m^−2^, respectively. It is clear that the Pt nanoparticles on the inner tube wall of SiO/SiO_2_ and Ti/SiO_2_ microtubes are larger than those on the inner tube wall of Ti/Co and SiO_2_/Ti microtubes, but the densities show the opposite result.

### The Motion of Pt Nanoparticle-Decorated Tubular Microengine

**Fig. 3:**
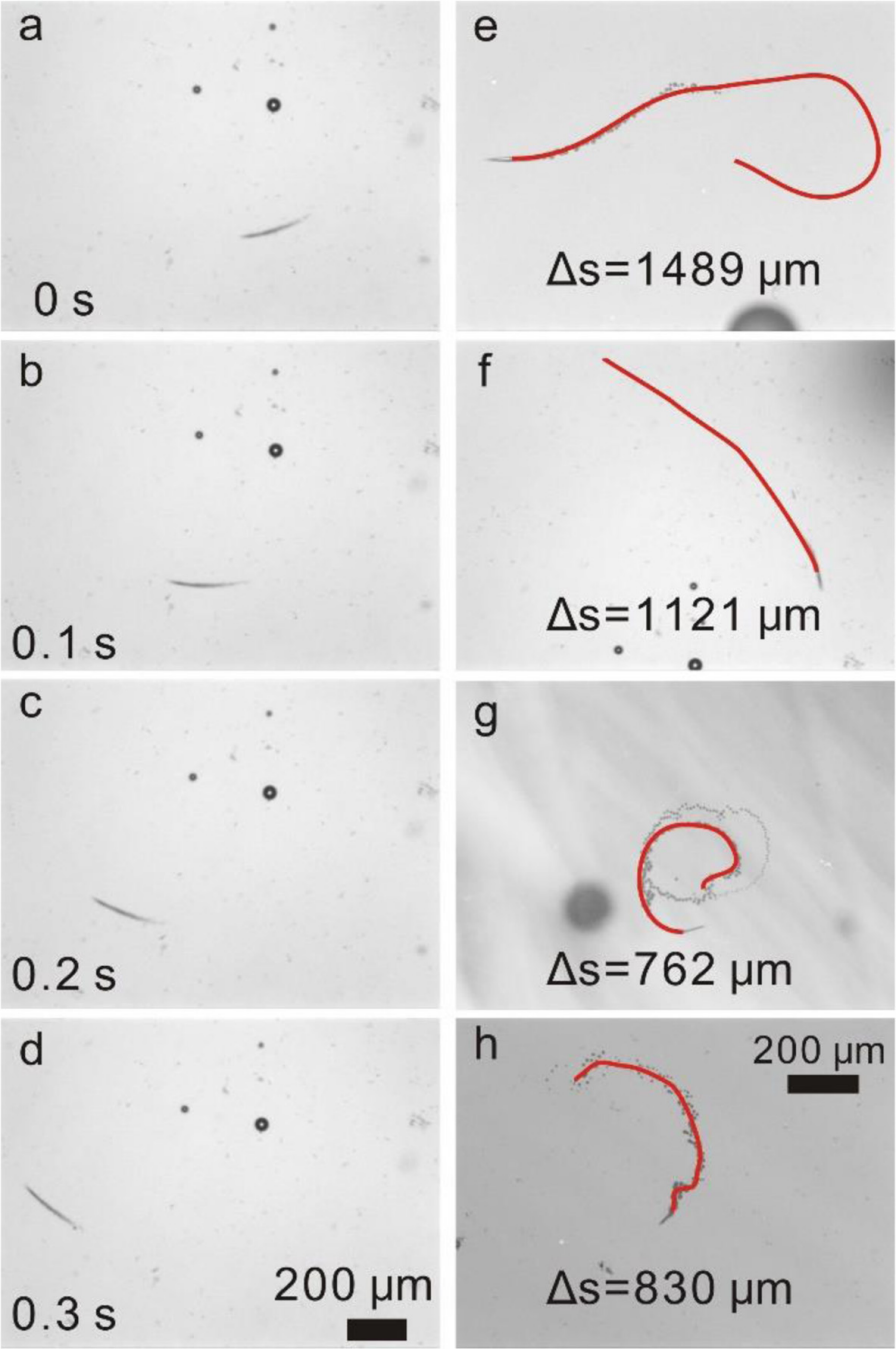
**a**–**d** Selected motion images of SiO/SiO_2_/Pt microengines at **a** 0, **b** 0.1, **c** 0.2, and **d** 0.3 s in 10 % H_2_O_2_ solution. **e**–**h** Trajectories of the four microengines decorated with Pt nanoparticles: **e** SiO/SiO_2_, **f** Ti/SiO_2_, **g** Ti/Co, and **h** SiO_2_/Ti. The *red* trajectories were recorded over a time period of 0.5 s in 10 % H_2_O_2_

Figure 3a–d shows time-lapse images of the movement of Pt nanoparticle-decorated SiO/SiO_2_ microengines in 10 % H_2_O_2_ (see also Additional file 2: Video 1). Oxygen bubbles ejected from one large end of microengine through the decomposition of H_2_O_2_ and propelled the microengine in opposite direction [40]. It is worth noting that both inner and outer surfaces are covered with Pt nanoparticles after Pt coating by ALD, but we observed no O_2_ bubbles generating on the outer surfaces of microengines. This indicates that O_2_ molecules have different nucleation behaviors on the inner and outer surfaces. The similar phenomenon had also been found in single-component metal oxide tubular microengines controlled by UV light [41]. It is considered that the geometries of the microengines have significant influence on bubble nucleation and generation. Generally, the bubbles can be formed on solid surfaces, if the gases reach heterogeneous nucleation energy [42]. Previous literature demonstrated that there are two factors determine the heterogeneous nucleation energy: the gas saturation concentration and the curvature of the surface [43]. The energy required for bubble formation on a flat surface is less than on a convex surface, and even less energy is required on a concave surface. It indicated that the gas produced on the concave surface of inner tube wall is much easier to nucleate compared than that on the convex surface of outer tube wall. In addition, different from other microengines such as Janus–motor [44] and Au-Pt nanorod [45], our microtubes can be used as a gas collecting chamber and O_2_ molecules produced inside the microtube will easily reach the supersaturation concentration for the bubble nucleation due to the accumulative effect of the inner confined space [46]. The accumulated O_2_ gas in the microtube can further facilitate the bubble nucleation. We noticed that the existence of Pt nanoparticles on the surface of the tube wall makes the catalytic decomposing reaction much more intense compared with smooth Pt layer, and high frequency bubble generation forms a long tail at the tube end. In our previous work [47], for a microtubular engine, we used the following Eq. (1) to calculate the oxygen productivity *dV*_O2_/d*t*:

1 $$ \mathrm{d}{V}_{{\mathrm{O}}_2}/\mathrm{d}t=n{C_{{\mathrm{H}}_2}}_{{\mathrm{O}}_2}\pi \mathrm{R}\mathrm{L} $$

where *n* is O_2_ production rate constant which was experimentally estimated to be ≈9.8 × 10^−4^ ms^−1^ in our previous work [46] and ${C_{{\mathrm{H}}_2}}_{{\mathrm{O}}_2}$ is the concentration of H_2_O_2_. This equation is considered to be valid for the microengines with a smooth inner surface. However, for the current microengines decorated with Pt nanoparticles, the inner surface area is much bigger than 2*π*RL. Apparently, the oxygen production is much higher than the microengines with smooth Pt layer, suggesting that Pt nanoparticle-decorated microengines can produce more oxygen. The corresponding bubble generation frequency makes the decorated microengines move in higher speed as we will explain in detail later. Detailed analysis of the video and time-lapse images demonstrates that the microengine was propelled at an ultrafast speed of around 3200 μm s^−1^ (Additional file 2: Video 1). According to the literature, for swimmer at low Reynolds number, the drag force (*F*) acted on the microengine is proportional to the motion speed (*v*) [47],

2 $$ F=-\frac{2\pi \mu Lv}{1\mathrm{n}(X)-0.72} $$

where *X* = 2 L/R is a geometrical parameter (*L* and *R* are the length and radius of the microengine, respectively) and *μ* is the fluid viscosity. The motion with faster speed means that the Pt-coated microengines need to overcome higher resistance. Moreover, the output power is proportional to the square of the motion speed since the output power is the product of the driving force and speed. In present case, one can deduce that the output power of Pt nanoparticle-decorated Ti/SiO_2_ microengines is also remarkably increased due to its ultrafast speed, and quantitative analyses of the speed promotion will be given below. We believe that the higher output power could enable this kind of microengines to accomplish more complex tasks in the future. For instance, we observed a powerful microengine spewing tiny bubbles off their back and push along a big bubble in the front (see the Additional file 3: Video 2), suggesting potential applications of powerful microengines in the field of microdelivery [48] or smart drug delivery [49]. It is worth noting that the performance enhancement is not limited to SiO/SiO_2_ microengine after decoration with Pt nanoparticles. Our results indicate that Pt nanoparticle decoration also leads to acceleration of other kinds of microtubular engines. In order to elucidate this phenomenon clearly, in Fig. 3e–f, we show the trajectories of four microengines moving in 10 % H_2_O_2_, extracting from the corresponding Additional files 4, 5, 6, and 7: Videos 3–6. One may note that the trajectories and the microbubble tails show unique geometries like linear, circular, and helical curves (Additional file 1: Figure S2). It is considered to be due to the imperfection in the microtubular structures, which generates a torque which is not parallel to the axis of microtubes resulting in different movement behaviors [40]. Quantitatively, the moving distances over a period of 0.5 s are 1489, 1121, 762, and 830 μm for Pt nanoparticle-decorated SiO/SiO_2_, Ti/SiO_2_, Ti/Co, and SiO_2_/Ti microtubes, respectively (Fig. 3). We found that particle distribution and size have a great influence on the surface area and therefore the performance of microengines. The surface area of SiO/SiO_2_/Pt, Ti/SiO_2_/Pt, Ti/Co/Pt, and SiO_2_/Ti/Pt microengines is 1.48, 1.80, 1.42, and 1.20 times larger, respectively, compared with smooth microtubular structure (Additional file 1: Figure S3), and thus, they demonstrated efficient catalytic effect, powerful propulsion thrust, and distinct moving trajectories, as shown in the Additional files 8, 9, 10 and 11: Videos 7–10. In addition, the enhanced surface area due to the existence of Pt nanoparticles also makes the microengines available to work in solution with low H_2_O_2_ concentration, and the motion of Pt nanoparticle-decorated microengines in 5 mL 10 % H_2_O_2_ after 24 h was shown in Additional file 12: Video 11. We experimentally found that the threshold H_2_O_2_ concentration for current Pt nanoparticle-decorated microengine can be as low as ~0.5 %. The time-lapse images in Additional file 1: Figure S4 display a Pt nanoparticle-decorated SiO/SiO_2_ microengine moving in a 0.5 % H_2_O_2_ solution. Although the oxygen bubble generation frequency is low, the microengine is nonetheless self-propelled at a speed of ~100 μm/s.

### The Experimental Results and Theoretical Model

**Fig. 4:**
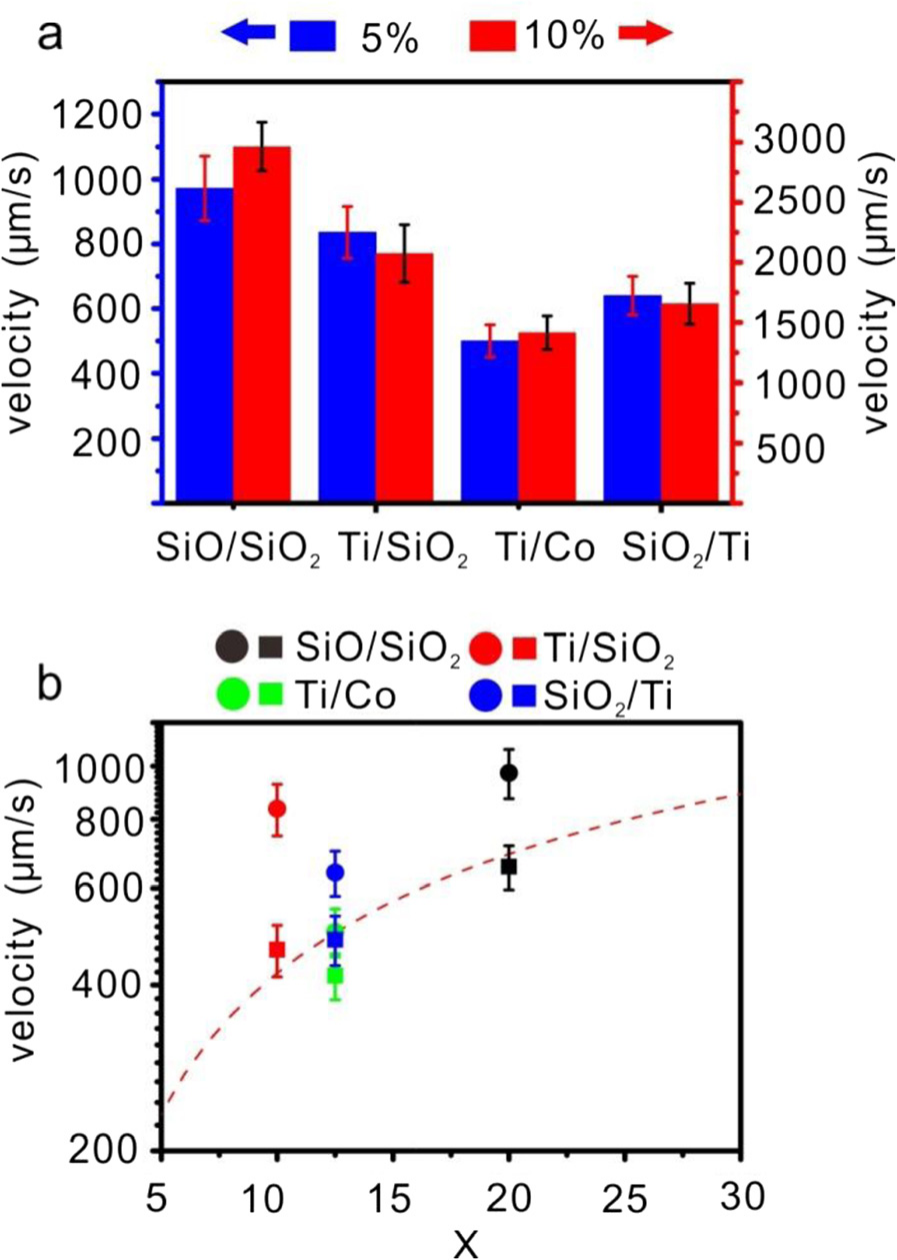
Average speed of catalytic microengines decorated with Pt nanoparticles. **a** Average speeds of the four types of Pt nanoparticle-decorated microengines moving in 5 and 10 % H_2_O_2_ solutions, respectively. **b** The dependence of the average speed of microengines on the tube geometric parameter (*X* = 2 L/R) in a 5 % H_2_O_2_ aqueous solution. The *red dashed line* is from the theoretical prediction. The results from the experiment are demonstrated by *colorized circles*, and *colorized squares* represent the speeds after the surface areas are normalized

To investigate the motion of decorated microengines in more details, we have calculated average speed of the four types of microengines based on statistics of 10 microengines in each case. Figure 4a shows the average speeds of the four types of Pt nanoparticle-decorated microengines moving in 5 and 10 % H_2_O_2_ solution. It is obvious that the average speeds of all four types increase with the concentration of H_2_O_2_ due to higher O_2_ productivity (see below).

According to body deformation model [45], the bubbles propel the microengine in a stepwise manner and the average speed of the smooth tubular microengines (*v*) can be theoretically predicted from Eq. (3)

3 $$ v=\frac{9n{C}_H{{{}_{{}_2}}_O}_{{}_2}X}{6+X/\left(1\mathrm{n}X-0.72\right)}, $$

The above equation suggests that the average speed of a microengine is mainly determined by the geometrical parameter X under the certain H_2_O_2_ concentration, as plotted by the red dashed curve in Fig. 4b. One can see that the Pt nanoparticle-decorated SiO/SiO_2_, Ti/SiO_2_, and SiO_2_/Ti microengines exhibit higher speeds compared with the theoretical prediction (1.38, 2, and 1.18 times, respectively), mainly due to the increase in the surface areas. If the surface areas are normalized (red, blue, and black squares in Fig. 4b), the experimental results can fit theoretical prediction very well if one notices that the surface areas were calculated by a simple approach (Additional file 1: Figure S3). This further proves that the larger surface area of the Pt nanoparticles (as calculated before) is mainly responsible for the highly efficient propulsion behavior of microengines, although the nanoparticle geometry may also affect the catalytic activity [32, 33]. Whereas in the case of Pt nanoparticle-decorated Ti/Co microengine (green square in Fig. 4b), the motion speed is slower than the theoretical prediction if the surface area is normalized. The surface area increased 1.42 times, but the speed increased only 1.26 times compared to the theoretical calculation. We assign this deviation to different surface morphology: the surface of the Ti/Co is unflat compared to other three samples, especially those with pure oxide bilayer nanomembrane, as we have mentioned above (see Fig. 2g). This may significantly influence the nucleation of gaseous microbubbles in the tubular chamber during catalytic motion and may also influence the dynamics of the microengine when it moves with high speed at low Reynolds number. In addition, we cannot rule out the possibility of the existence of electrochemical process in the O_2_ production. The Ti/Co microengine is the only one in the current four samples with conductive tube wall. Although this needs further investigation, we consider that the electrochemical process therein may be one of the possible reasons leading to smaller O_2_ productivity and corresponding slow motion speed.

## Conclusions

We have demonstrated a convenient method to produce modified microtubular structures for high-speed microengines by employing ALD of Pt nanoparticles. Experimental results demonstrated that Pt nanoparticles coated on the walls of microtubes enabled a dramatic enhancement of the catalytic reaction and correspondingly acceleration of motion speed due to increased surface area. The efficient propulsion performance of microengines holds considerable promise for catalysis support, drug/gene delivery, and medical imaging/diagnostics.

## Methods

### Fabrication of Microtubular Structures

Rolled-up microtubes consisting of different bilayer nanomembranes were prepared on polymer sacrificial layers. The 3510 T photoresist was spun coated on silicon substrate for 9 s at 600 rpm and 30 s at 3000 rpm then baked at 100 °C for 1 min, plus 10 min cooling down in the air. The resist got exposed in the mask-aligner for 10 s after the photomask has been aligned, and then resist was developed for 30~60 s. Ti/Co, SiO/SiO_2_, SiO_2_/Ti, and Ti/SiO_2_ bilayers of 10/10, 5/20, 10/20, and 20/10 nm, respectively, were then deposited on photolithographically patterned circles and squares via e-beam evaporation. The samples were deposited with different rates (i.e., 1/1, 5/0.5, 1/2, and 2/1 Å s^−1^, respectively) to build a strain gradient in nanomembrane under a high vacuum of 3.0 × 10^−4^ Pa. The samples were put in different angles inclined relatively to the horizontal to open an etching window at the far end of patterns. The intrinsic strain gradients in the bilayers after removing sacrificial photoresist layer by acetone made the bilayers roll into microtubular structures. To avoid collapse caused by the surface tension of the etchants, the samples were then dried in a critical point dryer (Leica CPD 030) using liquid CO_2_ as the intermedium.

### Pt Nanoparticle Deposition

Seventy cycles of Pt were deposited on the inner and outer surfaces of the prepared microtubes by ALD in a fluidized bed reactor. During the ALD process, (MeCp)Pt(Me)_3_ and oxygen were used as precursors. Herein, the precursors (MeCp)Pt(Me)_3_ and O_2_ were pulsed into the reaction chamber by the carrier gas argon, and the temperature was kept at 70 °C. During the ALD process, the working pressure in the chamber was maintained at 5 mbar.

### Motion Characterization

H_2_O_2_ solutions with different concentrations as fuel sources were added to activate the microengines at room temperature. An optical microscope (Olympus BX51) with an integrated camera was adopted to observe movement and locomotion of the microengines at a rate of 30 frame s^−1^. With the assistance of Image J, a detailed investigation of trajectories and speed was carried out.

## Additional files

Additional file 1: Figure S1. Pt nanoparticle size distribution on four different samples: **a** SiO/SiO_2_ nanomembrane, **b** Ti/Co nanomembrane, **c** Ti/SiO_2_ nanomembrane, and **d** SiO_2_/Ti nanomembrane. **Figure S2.** Propulsion images showing the motion trajectories and corresponding tracking lines of Pt nanoparticle-decorated microengines. The right schematic diagrams sketch the corresponding force analysis of microengines. Movements: **a** circular and self-rotation, **b** helical, **c** linear, and **d** snake-like motions. Scale bars 200 μm. **Figure S3.** The SEM image of Pt nanoparticles on SiO/SiO_2_ nanomembrane after being processed with the software Image J. **Figure S4.** Time-lapse images of a moving Pt nanoparticle-decorated SiO/SiO_2_ microengine in solution with low H_2_O_2_ concentration of 0.5 %. The interval of each image is 0.1 s. (DOCX 525 kb) Additional file 2:
**Video 1.** (AVI 1578 kb) Additional file 3:
**Video 2.** (AVI 5877 kb) Additional file 4:
**Video 3.** (AVI 1283 kb) Additional file 5:
**Video 4.** (AVI 1571 kb) Additional file 6:
**Video 5.** (AVI 1731 kb) Additional file 7:
**Video 6.** (AVI 13004 kb) Additional file 8:
**Video 7.** (AVI 5991 kb) Additional file 9:
**Video 8.** (AVI 1910 kb) Additional file 10:
**Video 9.** (AVI 2853 kb) Additional file 11:
**Video 10.** (AVI 1708 kb) Additional file 12:
**Video 11.** (AVI 2158 kb)

## Abbreviations

ALD: atomic layer deposition
(MeCp)Pt(Me)_3_: methylcyclopentadienyl-(trimethyl) platinum(IV)
SEM: scanning electron microscopy
