# Supplementary material for: Atomic Layer Deposition of Pt Nanoparticles for Microengine with Promoted Catalytic Motion
Source: Nanoscale Res Lett. 2016 Jun 13;11:289. doi: 10.1186/s11671-016-1515-5 (PMC4905863; doi:10.1186/s11671-016-1515-5)
Supplement: Additional file 1: Figure S1. — Pt nanoparticle size distribution on four different samples: a SiO/SiO2 nanomembrane, b Ti/Co nanomembrane, c Ti/SiO2 nanomembrane, and d SiO2/Ti nanomembrane. Figure S2. Propulsion images showing the motion trajectories and corresponding tracking lines of Pt nanoparticle-decorated microengines. The right schematic diagrams sketch the corresponding force analysis of microengines. Movements: a circular and self-rotation, b helical, c linear, and d snake-like motions. Scale bars 200 μm. Figure S3. The SEM image of Pt nanoparticles on SiO/SiO2 nanomembrane after being processed with the software Image J. Figure S4. Time-lapse images of a moving Pt nanoparticle-decorated SiO/SiO2 microengine in solution with low H2O2 concentration of 0.5 %. The interval of each image is 0.1 s. (DOCX 525 kb) [file 11671_2016_1515_MOESM1_ESM.docx]

Supporting Information

**Atomic layer deposition of Pt nanoparticles for microengine with promoted catalytic motion**

Chi Jiang, Gaoshan Huang,* Shijin Ding, Hongliang Dong, Chuanling Men, Yongfeng Mei*

Chi Jiang, Prof. Chuanling Men

School of Energy and Power Engineering

University of Shanghai for Science and Technology

Shanghai 200093, China

Prof. Gaoshan Huang, Dr. Hongliang Dong, Prof. Yongfeng Mei

Department of Materials Science

Fudan University

Shanghai 200433, China

Email: gshuang@fudan.edu.cn; yfm@fudan.edu.cnn

Prof. Shijin Ding

School of Microelectronics

Fudan University

Shanghai 200433, China

S1．Size distribution of Pt nanoparticles.


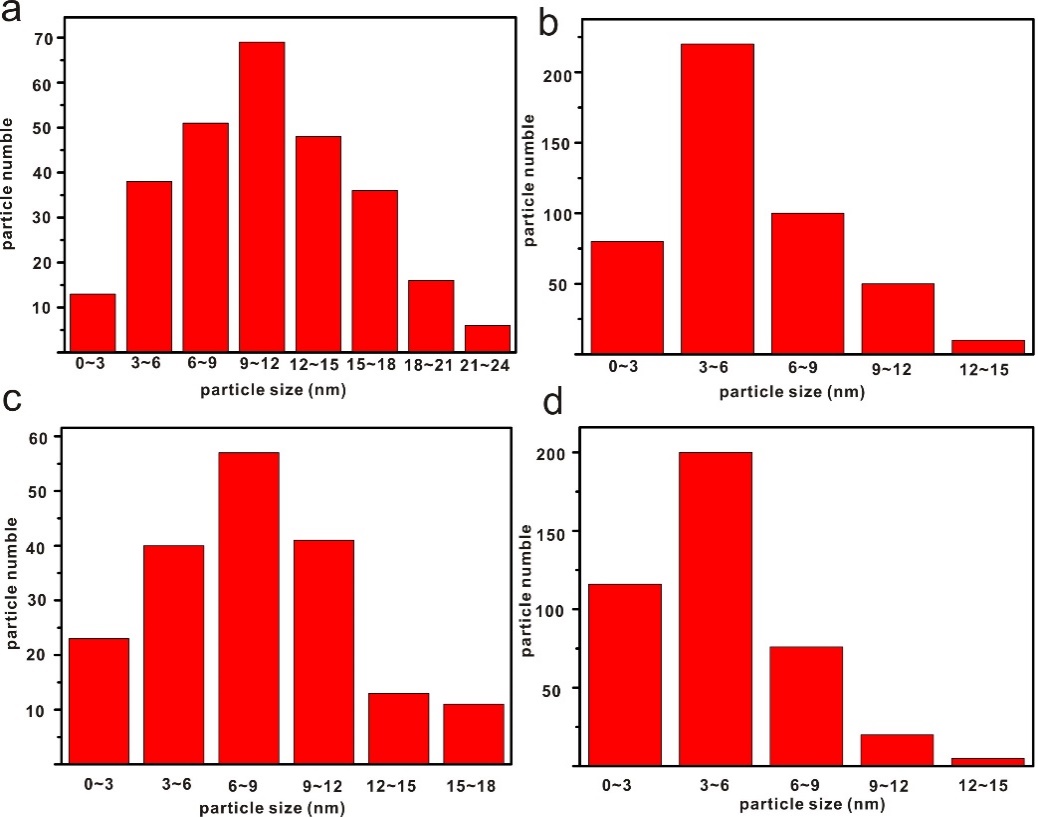


**Figure. S1** Pt nanoparticle size distribution on 4 different samples: a) SiO/SiO_2_ nanomembrane; b) Ti/Co nanomembrane; c) Ti/SiO_2_ nanomembrane; d) SiO_2_/Ti nanomembrane.

S2. Different motion behaviors of the microtubular engines.


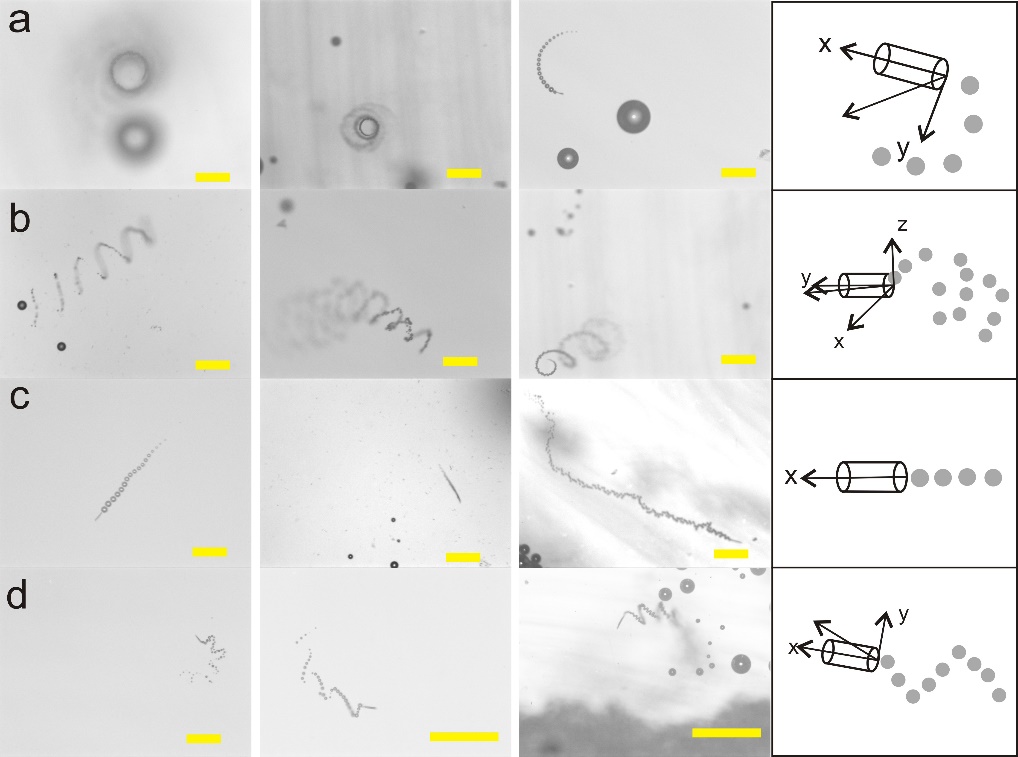


**Figure. S2** Propulsion images showing the motion trajectories and corresponding tracking lines of Pt nanoparticles decorated microengines. The right schematic diagrams sketch the corresponding force analysis of microengines. Movements: a) circular and self-rotation, b) helical, c) Linear, and d) snake-like motions. Scale bars: 200 μm.

The optical images in **Figure S2** show the different moving trajectories of Pt nanoparticles decorated microengines. The bubbles spewing from one end of microengines lead to unique tracking lines with a) circular and self-rotation, b) helical, c) linear, and d) snake-like motions. The anisotropic distribution of drag forces along the axial and radial directions cause a torque which is not parallel to the axis of microengines. The right schematic diagrams sketch the corresponding force analysis of microengines. For linear motion, the resultant forces on the microengines are parallel to the axial direction. However, for circular, self-rotation and snake-like motion, there was an angle between resultant force and XY-axis direction. Microengines show a helical 3D motion due to a torque in the Z-axis direction. ( Martín A, Jurado-Sánchez B, Escarpa A, Wang J. (2015). Template Electrosynthesis of High‐Performance Graphene Microengines. Small 11: 3568.)

S3. Estimation of the surface areas.


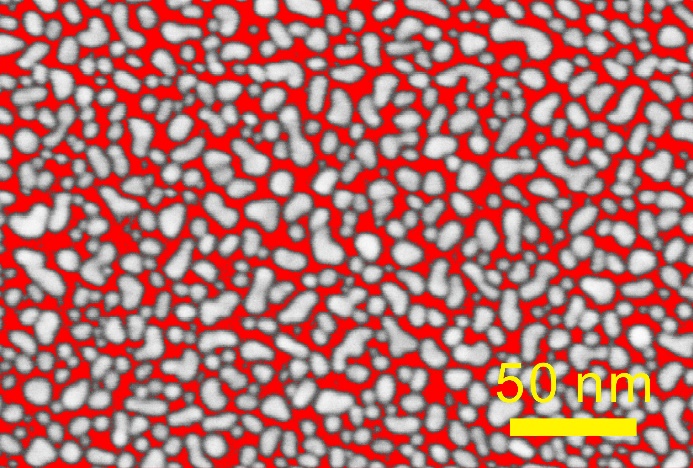


**Figure S3.** The SEM image of Pt nanoparticles on SiO/SiO_2_ nanomembrane after being processed with the software Image J.

In order to estimate the ratio of surface areas of Pt nanoparticles decorated nanomembrane and smooth surface, we used the following simplified approach. As shownin Figure S3, firstly, we calculated out the total projected area (the grey area, S_1_) of all nanoparticles in SEM image by using software **Image J**. For the sake of simplicity, we assume all the nanoparticles have the hemispherical shape. Thus, the projected area of a hemisphere is πR^2^ and its surface area is 2πR^2^. The total surface area of nanoparticles is 2S_1_. If the nanomembrane is smooth without nanoparticles the surface area (S_2_) should equal to the product of the length and width, which can be easily obtained from SEM image. The ratio of surface areas of Pt nanoparticles decorated nanomembrane and smooth surface (N) can be given by

$N=\frac{2S_{1}}{S_{2}}$.

For instance, we obtained the S_1_ and S_2_ for Pt nanoparticles decorated SiO/SiO_2_ nanomembrane are 6.1657×10^-14^ m^2^ and 8.3002×10^-14^ m^2^, respectively. So N is equal to 1.48. For Pt nanoparticles decorated Ti/SiO_2_, Ti/Co, SiO_2_/Ti nanomembranes, the ratios can be calculated in the same way and the results are 1.80, 1.20, and 1.42, respectively.

S4. Motion of microengine in solution with low H_2_O_2_ concentration.


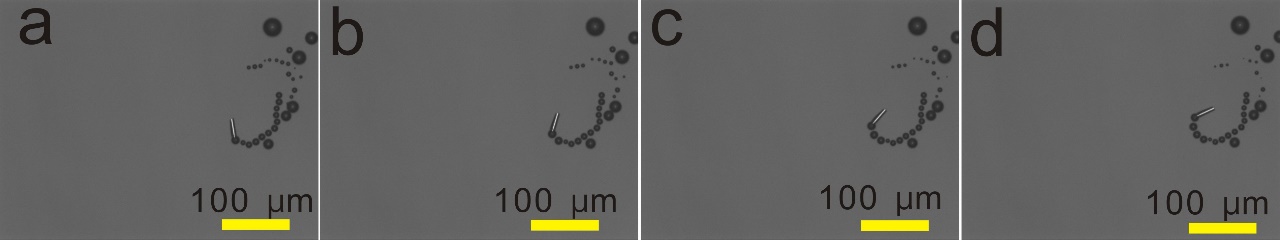


**Figure. S4** Time-lapse images of a moving Pt nanoparticles decorated SiO/SiO_2_ microengine in solution with low H_2_O_2_ concentration of 0.5%. The interval of each image is 0.1 s.
